# Autism-associated CHD8 deficiency impairs axon development and migration of cortical neurons

**DOI:** 10.1186/s13229-018-0244-2

**Published:** 2018-12-19

**Authors:** Qiong Xu, Yuan-yuan Liu, Xiaoming Wang, Guo-he Tan, Hui-ping Li, Samuel W. Hulbert, Chun-yang Li, Chun-chun Hu, Zhi-qi Xiong, Xiu Xu, Yong-hui Jiang

**Affiliations:** 1 Department of Child Health Care, Children’s Hospital of Fudan University, Shanghai, 201102 China; 2 Guangxi Key Laboratory of Regenerative Medicine & Guangxi Collaborative Innovation Center for Biomedicine, School of Preclinical Medicine, Guangxi Medical University, Nanning, 530021 Guangxi China; 3 Department of Pediatrics, Duke University School of Medicine, Durham, 27710 NC USA; 4 Department of Neurobiology, Duke University School of Medicine, Durham, 27710 NC USA; 5 Institute of Neuroscience & State Key Laboratory of Neuroscience, CAS Center for Excellence in Brain Science and Intelligence Technology, Shanghai Institutes for Biological Sciences, Chinese Academy of Sciences, Shanghai, 200031 China; 6 University of Chinese Academy of Sciences, Beijing, 100049 China; 7 Program in Genetics and Genomics, Duke University School of Medicine, Durham, 27710 NC USA; 8 Cellular Molecular Biology, Duke University School of Medicine, Durham, 27710 NC USA

**Keywords:** CHD8, Autism spectrum disorder (ASD), Chromatin remodeling, Neurite growth, Neurodevelopment

## Abstract

**Background:**

Mutations in *CHD8*, chromodomain helicase DNA-binding protein 8, are among the most replicated and common findings in genetic studies of autism spectrum disorder (ASD). The CHD8 protein is believed to act as a transcriptional regulator by remodeling chromatin structure and recruiting histone H1 to target genes. The mechanism by which deficiency of CHD8 causes ASD has not been fully elucidated.

**Methods:**

We examined the expression of *CHD8* in human and mouse brains using both immunohistochemistry and RNA in situ hybridization. We performed in utero electroporation, neuronal culture, and biochemical analysis using RNAi to examine the functional consequences of *CHD8* deficiency.

**Results:**

We discovered that CHD8 is expressed highly in neurons and at low levels in glia cells in both humans and mice. Specifically, CHD8 is localized predominately in the nucleus of both MAP2 and parvalbumin-positive neurons. In the developing mouse brain, expression of *Chd8* peaks from E16 to E18 and then decreases significantly at P14 to adulthood. Knockdown of *Chd8* results in reduced axon and dendritic growth, disruption of axon projections to the contralateral cortex, and delayed neuronal migration at E18.5 which recovers by P3 and P7.

**Conclusion:**

Our findings indicate an important role for CHD8 in dendritic and axon development and neuronal migration and thus offer novel insights to further dissect the underlying molecular and circuit mechanisms of ASD caused by CHD8 deficiency.

**Electronic supplementary material:**

The online version of this article (10.1186/s13229-018-0244-2) contains supplementary material, which is available to authorized users.

## Background

Autism spectrum disorder (ASD) is a group of clinically and molecularly heterogeneous neurodevelopmental conditions characterized by social communication deficits and restricted, repetitive behaviors [1, 2]. Genetic etiology has been implicated in approximately 20% of ASD patients. More than 70 genes or genomic loci have been confirmed as causative, and hundreds are considered strong candidates [3–12]. Of these, mutations in *CHD8* are among the most replicated and commonly identified genetic findings [6, 7, 13, 14].

CHD8 is a member of the chromodomain helicase DNA-binding protein family, which functions as an ATP-dependent chromatin remodeling factor and plays important roles in chromatin dynamics, transcription, and cell survival [15]. CHD8 is initially identified as a negative regulator of the Wnt–catenin signaling pathway through promoting the association of β-catenin and histone H1 and forming a trimeric complex on chromatin [16–18]. Accordingly, Wnt–β-catenin signaling was downregulated in *Chd8* mutant mice [18]. Homozygous deletion of *Chd8* in mice results in early embryonic lethality as a consequence of massive apoptosis in some lines of mutant mice [19, 20]. Haploinsufficiency of *CHD8* causes abnormal activation of RE-1 silencing transcription factor (REST), which suppresses the transcription of many neuronal genes. Moreover, CHD8 interacts physically with REST in the mouse brain [21]. Knockdown of *Chd8* by RNAi during cortical development results in defective neural progenitor proliferation, differentiation, and enhanced migration [18, 22]. Deficiency of *Chd8* in mice also alters synaptic transmission in striatal circuitry and results in functional over-connectivity in cortical networks [23]. Interestingly, a recent report also shows sexually dimorphic phenotypes related to abnormal neuronal excitation, enhanced inhibitory synaptic transmission and neuronal firing, and gene dysregulation in a new line of *Chd8* mutant mice [24]. Conditional knockout of *Chd8* in oligodendrocytes results in defective myelination in mice [25]. However, whether CHD8 deficiency affects axonal growth has not been studied.

Here, we report that CHD8 is specifically expressed in both excitatory and inhibitory neurons and to a lesser extent in GFAP+ astrocytes of cerebral cortex in human and mouse brains. Similar to a previous report, we found that the expression of *Chd8* peaks during embryonic development and decreases to relatively low levels postnatally in mouse brains [22]. *Chd8* deficiency in neocortex by RNAi using in utero electroporation resulted in delayed migration of cortical neurons and impaired dendritic and axonal growth. Our findings provide additional insights into the molecular and circuitry mechanisms underlying ASD caused by CHD8 deficiency. Defective axonal development may support a mechanism underlying the abnormal long distance connectivity reported in neuroimaging studies of ASD in humans.

## Methods

### Immunostaining of human brain sections

For light microscopic immunohistochemistry (IHC), human brain sections (30 μm) were incubated in 0.01 M phosphate-buffered saline (PBS) supplemented with 3% hydrogen peroxide for 10 min to block endogenous peroxidase and then in a blocking buffer containing 5% BSA/10% normal goat serum/0.25% Triton X-100 for 60 min at room temperature to prevent nonspecific staining. Following this, IHC was conducted on these free-floating sections and staining was visualized with a standard ABC Elite kit (Vector Labs).

For confocal microscopic double-labeling immunofluorescence, brain sections were incubated in the blocking buffer for 60 min at room temperature and then in a solution containing primary antibodies from different species simultaneously for one night at 4 °C. After washing, sections were incubated with appropriate secondary antibodies from Alexa Fluor series (Invitrogen, USA) for 45 min at 37 °C and then counterstained with Hoechst 33342 (1:5000; #C10022, Beyotime) for 15 min at room temperature to identify cellular nuclei. After mounting the sections, we observed them under a fluorescent confocal microscope (A1R Nikon).

Primary antibodies used were as follows: anti-CHD8 (1: 500; catalog #A301-225A; Bethyl Laboratories, Inc.), anti-GFAP (1:1000, catalog #MAB3402, Millipore), anti-MAP2 (1:2000, catalog #M9942, Sigma), anti-parvalbumin (Pvalb) (1:500, catalog #MAB1572, Millipore), and anti-DCX antibodies (1:200, catalog #Sc-8066, Santa Cruz).

For Nissl staining, Neuro Trace 500/525 green fluorescent Nissl stain (N21480) was bought from Thermo Fisher Scientific and used according to the manufacture’s instruction.

### Confocal microscopic double-labeling immunofluorescence of mouse brain slices

Mice were sacrificed by cervical dislocation at postnatal day 3 (P3) or P7. Their brains were removed and fixed in 4% (wt/vol) paraformaldehyde (PFA) overnight. After sequential dehydration in 15% and 30% sucrose at 4 °C, 50-μm coronal brain sections were cut with a cryostat, fixed in 4% PFA for 20 min at 4 °C, washed three times with PBS, blocked with 5% BSA and 0.3% Triton X-100 in PBS for 1 h at room temperature, and then incubated with rabbit anti-GFP (1:1000, catalog #A11122, Invitrogen) primary antibody overnight at 4 °C. The rinsed sections were then incubated with Alexa 488-conjugated goat anti-rabbit secondary antibody (1:3000; catalog #A-11122; Invitrogen) for 2 h at 37 °C. For endogenous CHD8 and NeuN immunostaining, sections were incubated with anti-CHD8 antibody (1: 500; catalog #A301-225A; Bethyl Laboratories, Inc.) and anti-NeuN antibody (1:500; catalog # MAB377; Millipore).

Cultured neurons were fixed with 4%PFA and then immunostained with primary antibodies against GFAP (1:1000, catalog #MAB3402, Millipore), MAP2 (1:2000, catalog #M9942, Sigma), and Tau1 (1:3000, catalog #MAB3420, Millipore) overnight at 4 °C. After washing, sections were then incubated with Alexa 488-conjugated goat anti-rabbit secondary antibody (1:3000, catalog #A-11122, Invitrogen) and Alexa 568-conjugated goat anti-mouse secondary antibody (1:2000, catalog #A-11004, Invitrogen) for 2 h at 37 °C.

### RNA extraction and real-time PCR

Forebrain tissues from embryonic or postnatal mice (C57BL/6) at different developmental stages (E16, E18, P0, P7, P14, P21, and adult) were used for RNA extraction. Mice were anesthetized with sodium pentobarbital and sacrificed via cervical dislocation, and brain tissues were quickly removed, dissected on ice, and homogenized with TRIzol Reagent (Invitrogen) at 4 °C. RNA was extracted according to the recommendations of the manufacturer, the final RNA pellet was suspended in diethylpyrocarbonate (DEPC)-treated water, and 2 μg of total mRNA was further subjected to reverse transcription using oligo (dT) primers and Moloney murine leukemia virus (M-MLV) transcriptase (Invitrogen). Real-time PCR was done with a LightCycler 480 Real-Time PCR System (Roche) according to the manufacturer’s instructions. Starting RNA levels were quantified by using GAPDH as the external standard. Primer sets were chosen from Primerbank, and gene sequences are available from the GenBank database. The primer sequences to examine the mRNA expression of mouse *Chd8* were as follows: forward, 5′-AAGCCCAGGTAACTCAAC-3′; reverse, 5′-TTCACATCGTCGGCGTCT-3′; GAPDH: forward, 5′-GGTTGTCTCCTGCGACTTCA-3′; reverse, 5′-CCACCACCCTGTTGCTGTAG-3′. The primers were synthesized by Invitrogen.

### RNA in situ hybridization

RNA in situ hybridization on the mouse brain sections was performed with digoxigenin (DIG)-labeled RNA probes. Full-length cDNA of *Chd8* was amplified with specific PCR primers and cloned into a pGEM-T easy vector (Promega) to generate an antisense probe for *Chd8*. The DIG-labeled antisense probes were synthesized by in vitro transcription using SP6 RNA polymerase. Mice of different developmental stages were perfused with 4% PFA, and fixed brains were sectioned into 20-μm slices using a cryostat. RNA in situ hybridization was performed as described previously [26]. Brain sections were hybridized for 18 h at 60 °C. The hybridization signal was detected with anti-DIG–alkaline phosphatase Fab fragments (Roche) and nitro blue tetrazolium chloride (NBT) plus 5-bromo-4-chlor-indolyl-phosphate (BCIP) as color reaction substrates.

### Human *CHD8* cloning and mouse shRNA construct preparation

Human *CHD8* cDNA (*CHD8*, variant 1 - HaloTag® human ORF in pFN21A) was purchased by Promega (catalog: FHC12740). PCR products were cloned into the pCAGGS-IRES-EGFP vector [27]. Oligonucleotides targeting different sequences of mouse *Chd8* cDNA and a scrambled control oligonucleotide were designed, synthesized, and cloned into pSuper-basic (Oligoengine). The 21-bp target sequences were as follows: shRNA#1, 5′-GCT GGT GGA CTT GGT ATT AAT-3′; shRNA#2, 5′-GCT GCT GAT ACC TGT ATT ATC-3′; shRNA#3, 5′-GCT ACA AGA GAG AAC AAA TGA-3′; and shRNA-scramble, 5′-TTC TCC GAA CGT GTC ACG T-3′. All constructs were verified by DNA sequencing.

### Western blot analysis

For the analysis of the expression of CHD8 in the developing mouse cortex, cortices were dissected and homogenized as previously described [28]. For knockdown and rescue experiments, electroporated cortical neurons were cultured for 3–7 days and lysed in 1× protein loading buffer (50 mM Tris-HCl, pH 6.8, 2% SDS, 10% glycerol, 1% 2-ME, and 0.1% bromophenol blue). All protein samples were separated by 10% SDS-polyacrylamide gel electrophoresis (Bio-Rad) and blotted onto PVDF membrane (Millipore). Membranes were blocked with 5% non-fat milk in 0.05% Tween 20 at room temperature for 1 h and probed with rabbit anti-CHD8 (Bethyl Laboratories, Inc.), HRP-coupled mouse anti-actin (Santa Cruz Biotechnology) antibodies. The secondary antibody used for CHD8 was goat anti-rabbit IgG coupled to HRP (Santa Cruz biotechnology). Bands were visualized by enhanced chemiluminescence (TIANGEN). Band intensities were measured with ImageJ Software.

### Primary neuron culture and electroporation

Primary cortical neurons were prepared from brains of P0 C57 mice as described previously [28]. Before plating, neurons suspended in transfection medium (200 μl) were mixed with 1 μg pCAG-EGFP and 3 μg shRNA for immunocytochemistry. Then, neurons were transferred into a 2.0-mm electroporation cuvette (Fisher) and transfected by electroporation using the Amaxa Nucleofector apparatus.

### In utero electroporation

In utero electroporation was performed as described previously with a few modifications [27]. Briefly, pregnant mice at E14.5 were anesthetized with 0.7% sodium pentobarbital at a dose of 10 μl/g and subjected to abdominal incision to expose the uterus. For different experiments, a mixture of GFP, RNAi, and/or rescue constructs was prepared. Plasmids (about 1 μl) with 0.05% Fast Green (Sigma) were injected into the lateral ventricle of embryos through a glass micropipette. Electrical pulses were then delivered to embryos by gently clasping their heads with forcep-shaped electrodes connected to an ECM-830 square-pulse generator (BTX). Five 50-ms pulses of 30 V were applied at 1-s intervals. Uterine horns were repositioned in the abdominal cavity, and the abdominal wall and the skin were sutured. Postsurgery animals were maintained in a warm animal room (25 °C) with plenty of water and food. At different developmental stages, mice were perfused transcardially with 0.9% saline followed by 4% PFA in 0.1 M phosphate buffer (PB; pH 7.4), and the brains were removed and fixed in 4% PFA for another 24 h. Fixed brains were cryoprotected with 30% sucrose and then sectioned using a cryostat.

### Image acquisition and quantification

Images were obtained with a Nikon A1R inverted confocal microscope with × 20 (numerical aperture, 0.7) objectives. Each image was a composite constructed from a series of images taken throughout the *z* aspect of the neuron. Images of isolated cells or cortical neurons in regions with a low density of GFP transfected cells were chosen for illustration, and three-dimensional (3D) reconstructions were made to trace dendritic or axonal arbors for measurement of total dendritic or axonal length. Neurolucida software (MBF Bioscience) was used to generate tracings to measure neurite lengths. Sholl analysis, a quantitative analysis by counting the number of neurite intersections of concentric circles of gradually increased radius centered at the cell body, was performed with Neurolucida Explorer (MBF Bioscience). Image acquisition and data analysis were performed in a double-blind manner.

### Statistical analysis

For all statistical analyses, experimenters were blinded for genotypes of mice and the treatments of the animals or cells. Data are presented as mean ± SEM. The paired or unpaired Student’s *t* test, one-way ANOVA with Dunnett’s post hoc tests or Bonferroni’s tests, or two-way ANOVA with Bonferroni post hoc tests were used for data analyses by SPSS 13.0 software. Statistical significance was defined as *P* < 0.05.

### Animals

Except in utero electroporation, for which ICR mice were used, all other experiments were performed in C57BL/6 J mice. Pregnant mice undergoing in utero electroporation were individually housed in plastic cages, and when they delivered, they were housed with their pups.

### Human brain tissues

Frozen postmortem brain tissues of Brodmann 19 region from a 6-year-old child was obtained from the NIH human brain tissue bank at the University of Maryland (http://www.medschool.umaryland.edu/btbank/). The postmortem time was 19 h.

## Results

### CHD8 is predominantly expressed in neurons of human cerebral cortex

**Fig. 1:**
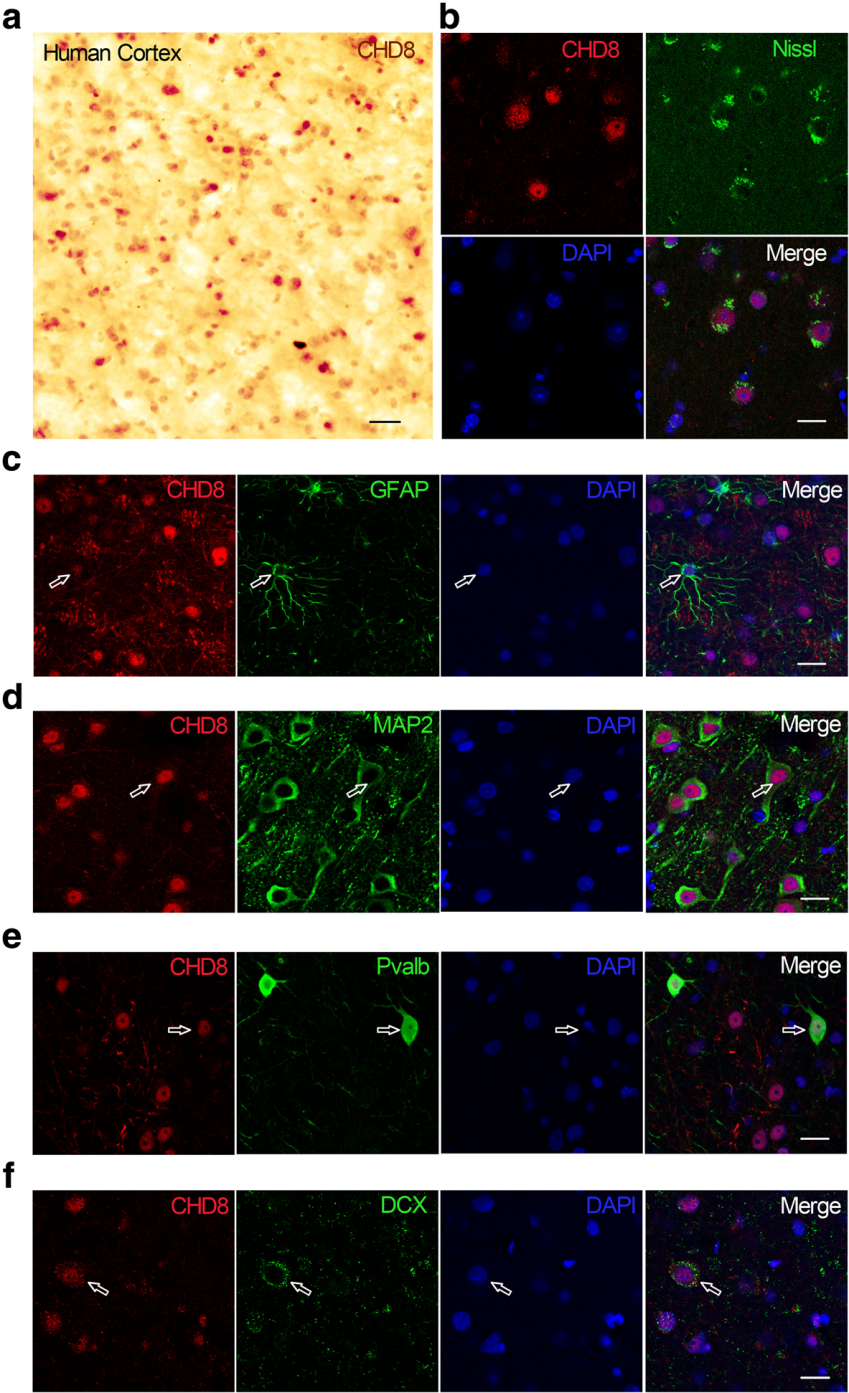
Expression of CHD8 in human cerebral cortex. **a** Immunohistochemical staining revealed the expression pattern of CHD8 in human cerebral cortex from Brodmann 19 region. **b** Human CHD8 is expressed in neurons. The sections were immunostained with CHD8 antibody and counterstained with NeuroTrace 500/525 green fluorescent Nissl (Thermo Fisher Scientific). **c** CHD8 is expressed in GFAP+ astrocytes (open arrows: astrocytes). **d** CHD8 is expressed in MAP2-positive neurons (open arrows for MAP2 positive neurons). **e** CHD8 is expressed in pavalbumin (Pvalb)-positive interneurons (open arrows for Pvab-positive interneuron). **f** CHD8 is expressed in DCX-positive neurons (open arrows for DCX+ neuron) (scale bar, 20 μm)

To probe the function of CHD8 and its pathophysiology underlying ASD, we first examined the expression pattern of CHD8 in a human brain sample. We performed immunohistological analysis using postmortem brain tissue (Brodmann 19) of a 6-year-old child. Strong staining of CHD8 was revealed in cerebral cortex (Fig. 1a). CHD8 was highly expressed in Neuro Trace Nissl stain-positive neurons but to a lesser extent in GFAP-positive glia cells (Fig. 1b, c). Consistent with the known function of CHD8 as a chromatin regulator, immunofluorescent staining showed that CHD8 was predominantly localized in the nucleus (Fig. 1b, f). Co-staining of CHD8 and neuronal markers showed that CHD8 was expressed in both MAP2-positive excitatory neurons **(**Fig. 1d) and parvalbumin (Pvalb)-positive interneurons (Fig. 1e). Moreover, CHD8 was expressed in Doublecortin (DCX)-positive neuronal precursor cells and immature neurons (Fig. 1f), which is consistent with the early onset of clinical manifestations in patients with *CHD8* mutations.

### *Chd8* is highly expressed in the developing mouse brain

**Fig. 2:**
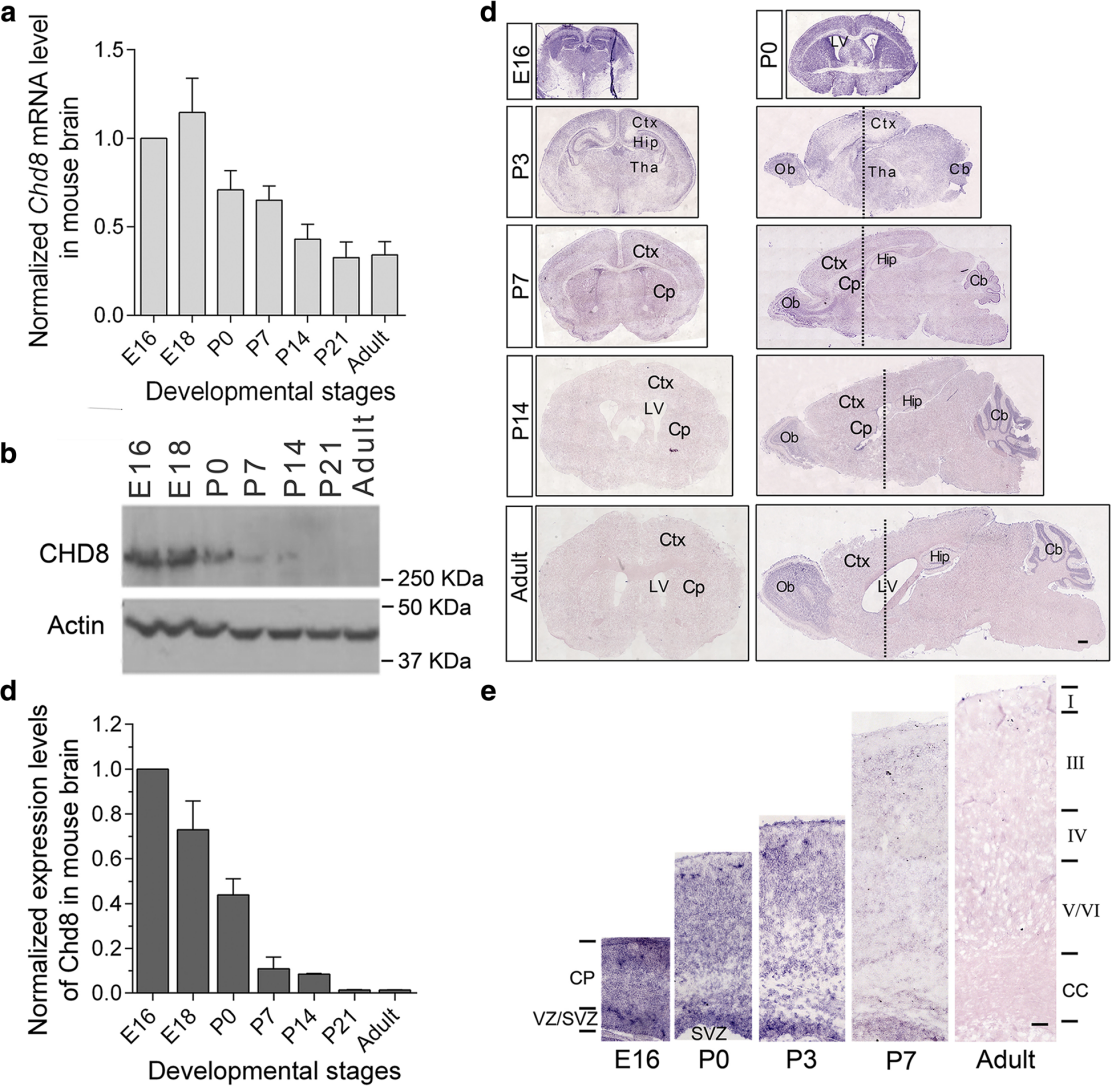
Expression of *Chd8* in the developing mouse brain. **a** *Chd8* mRNA expression in the developing mouse brain (*n* = 5 at each time point) by real-time RT-PCR. The level was normalized to the E16 mice. Error bars represent SEM. **b** Western blot analysis of CHD8 protein using whole brain lysates at the different developmental stages of mouse brains. **c** Quantification of CHD8 protein levels in developing mouse brain (*n* = 6 at each time point) by western blot analysis that is normalized to E16 level. **d** *Chd8* RNA in situ hybridization of sagittal and coronal sections from mouse brains at the stages indicated. Abbreviations: cortex (Ctx), lateral ventricle (LV), hippocampus (Hip), thalamus (Tha), olfactory bulb (Ob), cerebellum (Cb). Scale bar, 600 μm. **e** RNA in situ hybridization shows the *Chd8* expression pattern in coronal sections of the cerebral cortex at the stages indicated. Scale bar, 500 μm. High levels of *Chd8* mRNA are detected in the cortex at perinatal stages. Abbreviations: cortical plate (CP), ventricular zone (VZ), subventricular zone (SVZ), corpus callosum (CC). Roman numerals (I–VI) indicate layers of the cerebral cortex (scale bar, 100 μm)

**Fig. 3:**
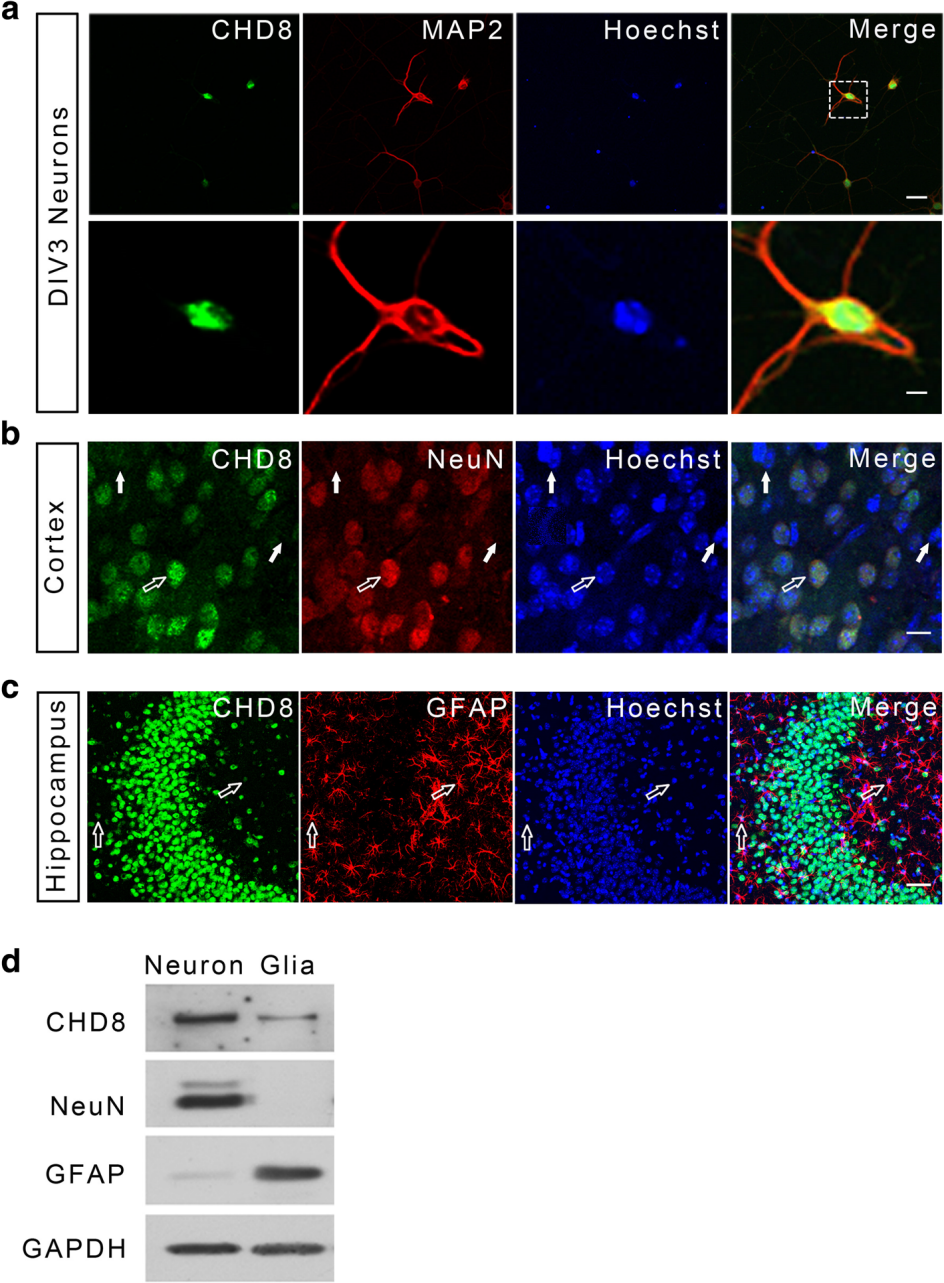
Cell type-specific expression pattern of CHD8 in mouse brains. **a** Endogenous mouse CHD8 is selectively enriched in the nucleus of cultured DIV3 cortical neurons. Cells were immunolabeled with CHD8 (green), the somatodendritic marker MAP2 (red), and with counterstaining of nuclear marker Hoechst (blue). Scale bar, 2 μm. **b** Double-immunofluorescent staining shows that CHD8 is highly expressed in NeuN-positive neurons. Scale bar, 5 μm. The open arrows indicate both CHD8 and NeuN-positive neurons, while the filled arrows indicate neither CHD8 nor NeuN-positive non-neuronal cells. **c** Double-immunofluorescent staining shows that CHD8 is weakly expressed in GFAP-positive astrocytes. The open arrows indicate GFAP-positive astrocytes with weak CHD8 expression. Scale bar, 25 μm. **d** Representative western blot shows that CHD8 is predominately expressed in neurons but also in cultured glia cells

Next, we examined the expression pattern of *Chd8* in the mouse brain at different developmental stages. Quantitative real-time mRNA expression analysis (qRT-PCR) (Fig. 2a) revealed that *Chd8* was detectable in neocortex from E16 to adulthood. The peak of the expression is at E18 and then markedly decreases at P7 and gradually declines to very low levels at adulthood. This expression pattern in neocortex was also confirmed at the protein level by western blot analysis (Fig. 2b, c). To examine the temporal and spatial expression pattern of *Chd8* in the developing brain, we performed RNA in situ hybridization on mouse brain sections. *Chd8* mRNA was most abundant in embryonic brain and then gradually reduced after birth, consistent with the results from the qRT-PCR and western blot analyses. Specifically, *Chd8* mRNA was present in the sub-plate (SP) and proliferative zones of the cortical wall (the ventricular zone [VZ] and the subventricular zone [SVZ]) at E16 and P0 and in the cortical plate (CP) of the cerebral cortex, hippocampus, striatum, cerebellum, and olfactory bulb from P3 to P7, a developmental period with extensive outgrowth and elaboration of dendrites and axons (Fig. 2d, e). The cellular and subcellular localizations of CHD8 in developing mouse brains and cultured neurons were also examined by immunostaining with neuronal cell markers. Consistent with the results in human cerebral cortex, mouse CHD8 was expressed in the neuronal nucleus, with a pattern similar to that of NeuN in neocortex and hippocampus in P3 mouse (Fig. 3a–c). CHD8 expression in neurons and glia cells was also confirmed by western blot analysis of cultured DIV3 neurons and glia cells of mice (Fig. 3d). However, the expression of CHD8 in glia cells was significantly lower than that in neurons. Taken together, the expression analyses of both human and mouse brains imply important roles for CHD8 during the development of cerebral cortex.

### CHD8 regulates neuronal morphogenesis in vitro

**Fig. 4:**
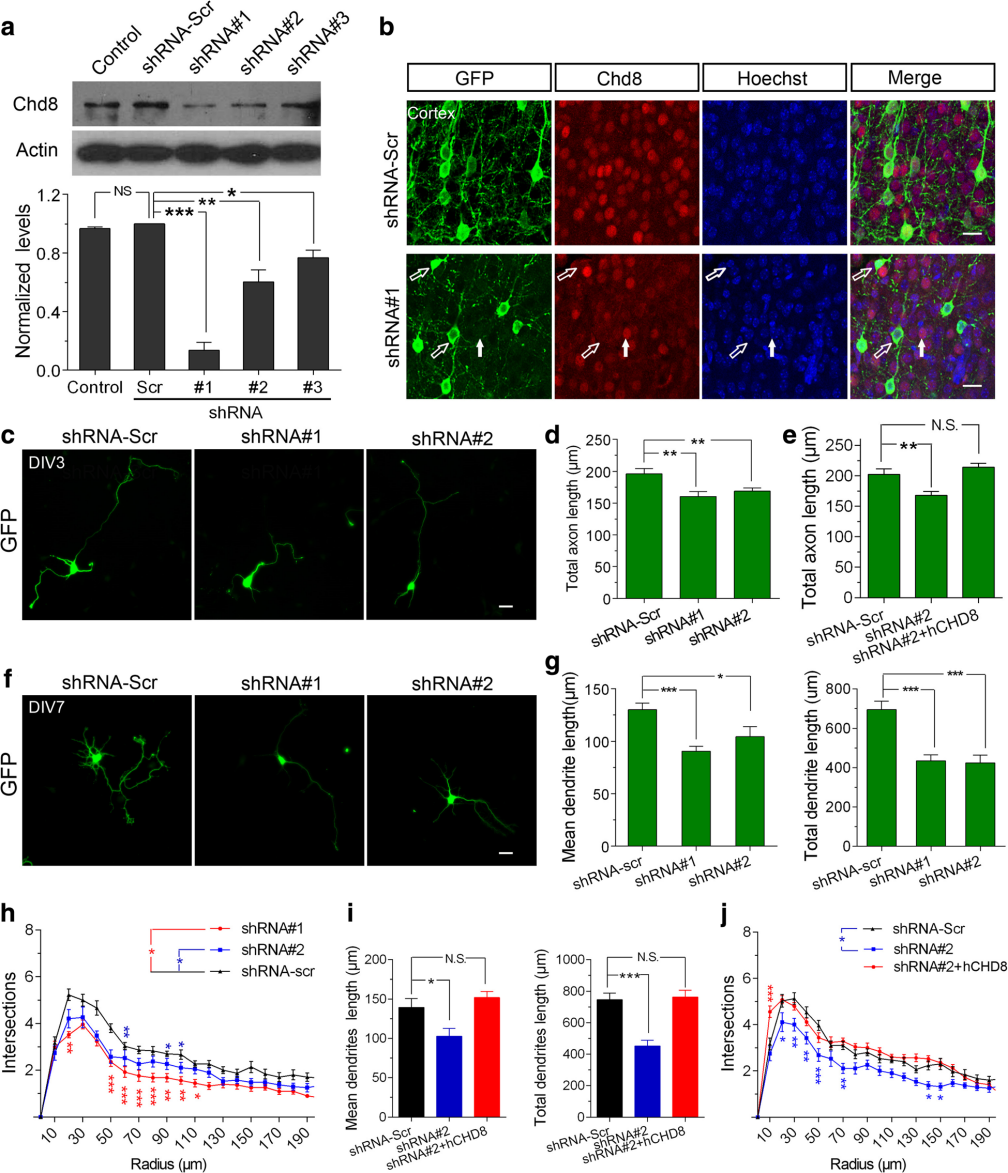
CHD8 regulates neurite growth in vitro. **a** shRNA-mediated knockdown of mouse *Chd8* in transfected cultured neurons. Upper, representative western blots; lower, quantification of western blot analysis. shRNA#1, 0.14 ± 0.095; shRNA#2, 0.60 ± 0.14; shRNA#1, 0.77 ± 0.089. For ANOVA, *P* = 0.587. For post hoc Dunnets’ test, **P = 0.004 for shRNA#1 vs shRNA-scr; *P = 0.041 for shRNA#2 vs shRNA-scr; *P = 0.046 for shRNA#3 vs shRNA-scr. All data were shown in mean ± SEM and were collected from three to six independent experiments. **b** Immunostaining showing shRNA-mediated knockdown of *Chd8* in transfected neurons in mouse brains. Immunohistochemical staining of endogenous CHD8 in coronal sections from a P0 mouse brain electroporated with shRNA#1 and GFP at E14.5. The open arrows indicate successfully transfected cells, and the filled arrows indicate normal cells. Scale bar, 10 μm. **c** Representative images of DIV3 cultured neurons transfected with GFP and the plasmids indicated. Constructs were transfected at DIV0, cultured for 3 days in vitro, and then immunolabeled with anti-GFP. Scale bar, 100 μm. **d** Quantification of the total axon length of neurons transfected with *Chd8* shRNA constructs. *n* = 126, 133, 124 neurons per group, respectively. Total axon length: shRNA-scr, 196.0 ± 8.42 μm; shRNA#1, 160.4 ± 7.56 μm. For ANOVA, *P* = 0.01. For post hoc Dunnets’ test, **P = 0.0032 for shRNA#1 vs shRNA-scr; shRNA#2, 168.8 ± 6.98, **P = 0.0052 for shRNA#2 vs shRNA-scr. **e** Defective axon growth in *Chd8* shRNA#2 construct was rescued in neurons by co-transfected with shRNA#2-resistant hCHD8 constructs (*n* = 50 neurons per group). Total axon length: shRNA-scr, 202.6 ± 9.13 μm; shRNA#2, 168.2 ± 6.68 μm. For ANOVA, *P* = 0.07. For post hoc Dunnets’ test, **P = 0.004 for shRNA#2 vs shRNA-scr; shRNA#2 + hCHD8, 214.4 ± 6.15, *P* = 0.32 for shRNA#2 + hCHD8 vs shRNA-scr. **f** Representative images of DIV7 cultured neurons transfected with GFP and *Chd8* shRNA constructs as indicated. **g** Quantification of dendritic length in neurons transfected with *Chd8* shRNA constructs as indicated. *n* = 80–100 neurons per group. Mean dendritic length: shRNA-scr, 130.1 ± 6.3 μm; shRNA#1, 90.7 ± 4.61 μm. For ANOVA, *P <* 0.001. For post hoc Dunnets’ test, ***P < 0.0001 for shRNA#1 vs shRNA-scr; shRNA#2, 104.5 ± 9.53, *P = 0.032 for shRNA#2 vs shRNA-scr. Total dendritic length: shRNA-scr, 694.9 ± 42.5 μm; shRNA#1 is 435.9 ± 30.6 μm. For ANOVA, *P* = 0.0007. For post hoc Dunnets’ test, ***P = 0.0001 for shRNA#1 vs shRNA-scr; shRNA#2 is 425.3 ± 39.2 μm, ***P = 0.0003 for shRNA#2 vs shRNA-scr. **h** Quantification of dendritic complexity as measured by Sholl analysis (*n* = 50–80 neurons per group). For ANOVA, *P* = 0.0015. For Bonferroni post-tests, *P < 0.05; **P < 0.01; **P < 0.001. **i** Co-expression of the human CHD8 rescued the defective dendritic growth by the shRNA-#2 in DIV7 neurons. *n* = 100–120 neurons in each group. Data represent mean ± SEM. For ANOVA, *P* = 0.0127 (left panel); *P* = 0.0013 (right panel). For post hoc Dunnett’s test, *P < 0.05; **P < 0.01; ***P < 0.001. **j** Quantification of dendritic complexity as measured by Sholl analysis (*n* = 50–80 neurons per group). For ANOVA, *P* < 0.001. For Bonferroni post-tests, *P < 0.05; **P < 0.01; ***P < 0.001

Altered short- and long-distance functional connectivity has been reported frequently in brains of human ASD patients by neuroimaging studies [29, 30]. Impaired dendritic development has been reported in numerous mouse models of ASD [31–33]. However, few studies have examined axonal development in ASD animal models. We therefore examined whether *Chd8* deficiency affects the development of both dendritic morphology and callosal axon growth during development using a shRNA approach. We designed three shRNA constructs that were targeting to *Chd8* and tested the efficiency in both cultured neurons and in vivo in mouse brains. Compared with the scramble shRNA construct, all shRNAs resulted in a significant decrease in the expression of CHD8 protein in cultured neurons, and among them, shRNA#1 had the highest efficiency (Fig. 4a). The efficacy of the shRNA constructs was confirmed in vivo in mouse brain (Fig. 4b). We also validated the rescued constructs against shRNA-mediated knockdown of mouse *Chd8* in transfected neurons in vitro (Additional file 1: Figure S1). To examine axonal growth, we co-transfected dissociated DIV0 mouse cortical neurons with plasmids encoding GFP and shRNA constructs by electroporation and then plated them to allow the neurons to grow in culture medium for 3 days (DIV3). We used Tau1 as an axon-specific marker. We found that depleting CHD8 significantly decreased total axon growth (Fig. 4c, d). However, co-expression of human CHD8 protein (hCHD8) that is shRNA#2-resistant was able to rescue the total axon length defects caused by shRNA#2 construct (Fig. 4c–e; Additional file 1: Figure S1). We next examined the effects of *Chd8* knockdown on dendritic outgrowth and branching in vitro. Control neurons were transfected at DIV0 with GFP plus a non-targeting shRNA construct (shRNA-scr). Seven days later, we analyzed the data after staining cells with the somatodendritic marker MAP2 (Fig. 4f). The total and mean dendrite length were significantly reduced in neurons expressing both shRNA#1 and shRNA#2 constructs compared to those expressing shRNA-scr (Fig. 4g). It is worth noting that the intersections were more affected by shRNA#1 construct than by shRNA#2 construct (Fig. 4h). This appears to be consistent with the more significant reduction of *Chd8* after shRNA#1 treatment than that of shRNA#2. The effects of shRNA#2 were rescued by co-transfecting shRNA2-resistant hCHD8 (Fig. 4i, j). These results indicate CHD8 is required for both axonal and dendritic development.

### CHD8 is essential for neurite development in vivo

**Fig. 5:**
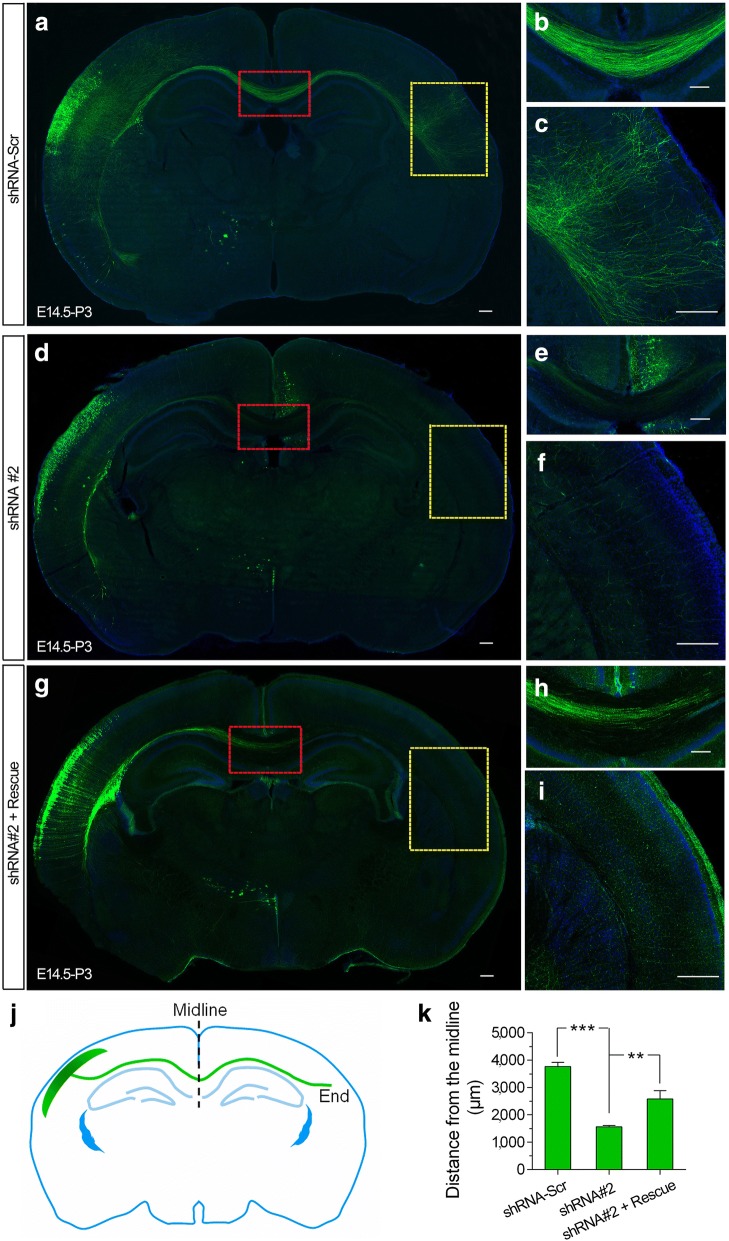
CHD8 regulates the axon growth in vivo. **a**–**i** Representative images of coronal slices of P3 mouse brains that were transfected with GFP together with indicated constructs by in utero electroporation at E14.5. High-magnification images are shown in the right. Brain slices at the level of Bregma − 1.58 mm were stained with Hoechst and GFP antibodies to visualize the callosal. Scale bar, 150 μm (**a**, **d**, **g**), 200 μm (**b**, **e**, **h**), or 400 μm (**c**, **f**, **i**). **j** A schematic for quantitative analysis of callosal axon length. The adjacent axon lengths were measured from the midline to end. **k** Quantitative analysis of callosal axon length *Chd8* ShRNA#2 construct showed significantly shorter axonal length than scramble control. The co-expression of human hCHD8 rescued the phenotypes caused by knockdown of *Chd8* by shRNA#2 construct. Distance from the midline: shRNA-scr, 3763 ± 154.3 μm; shRNA#2, 1563 ± 47.95 μm. ***P < 0.0001 shRNA#2 vs shRNA-scr; shRNA#-h*CHD8*, 2575 ± 307.2 μm. **P = 0.0021 shRNA#2-h*CHD8* vs shRNA-scr. Results are shown as mean ± SEM. *n* = 4 in each group. For ANOVA, *P* = 0.0016. For Bonferroni post-tests, **P < 0.01; ***P < 0.001

To test whether *Chd8* deficiency has effects on mouse brains in vivo, we used the shRNA#2 construct for this experiment because of the feasibility of conducting rescue experiments as demonstrated in vitro. At P3, mouse pups electroporated with scrambled shRNA had callosal axons originating from the electroporated neurons that crossed the midline and formed and arborized dense projections to the contralateral side of cerebral cortex, and the axon bundles were restricted to the corpus callosum (CC) (Fig. 5a–c, j, k). In contrast, in mice transfected with the *Chd8* shRNA#2 construct, the axon terminals were barely visible in the contralateral cerebral cortex, indicating significantly retarded axon growth and elongation (Fig. 5d–f, j, k). Co-transfection of the shRNA#2-resistant *hCHD8* construct successfully rescued these defects (Fig. 5g–k). These findings suggest that reduced expression of *Chd8* in callosal projection neurons impairs axon growth and branching in vivo.

**Fig. 6:**
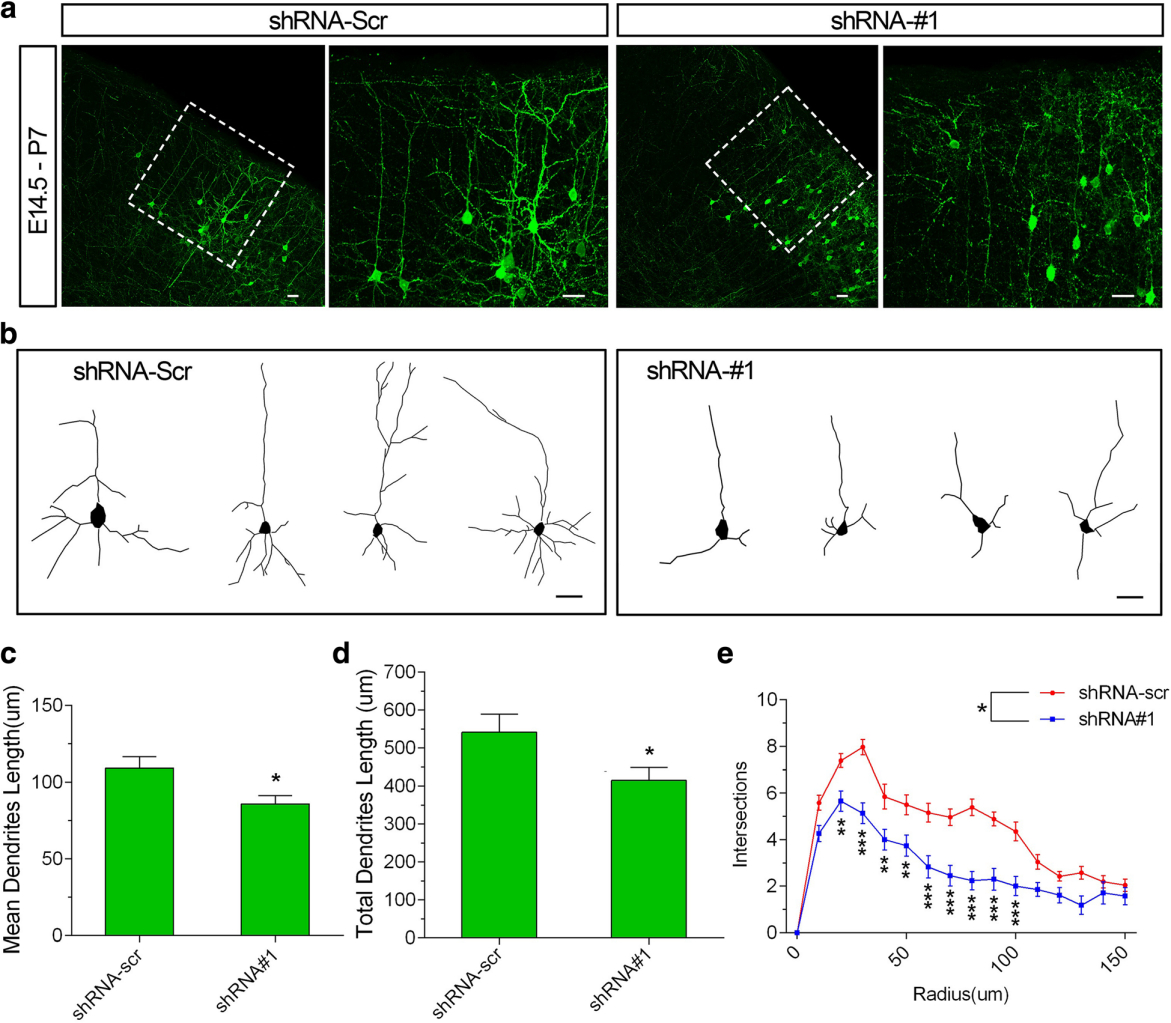
CHD8 is required for dendrite growth in vivo. **a** Representative images of coronal slices of P7 mouse brains that were transfected with GFP together with indicated constructs by in utero electroporation at E14.5. Transfected cells were visualized by staining coronal slices with GFP antibody. In each image pair, the right image is from the boxed region of the left image. Scale bar, 100 μm. **b** Neurolucida tracings of representative layer II/III pyramidal neurons, from experiment shown in **a**. Scale bar, 50 μm. **c**–**e** Quantitative analysis of dendrite length and dendrite complexity measured from Neurolucida tracings. Mean dendritic length: shRNA-scr, 109.2 ± 7.5 μm; shRNA#1, 85.9 ± 5.59 μm, *P = 0.015, shRNA#1 vs shRNA-scr. Total dendritic length: shRNA-scr, 541.6 ± 47.52 μm; shRNA#1, 415.233.89 μm, *P = 0.0332, shRNA#1 vs shRNA-scr. Greater than 70 neurons were examined in each group from at least three independent experiments. Results are presented as the mean ± SEM. *P < 0.05; **P < 0.01; ***P < 0.001; one-way ANOVA post Dunnett’s test

To assess the impact of *Chd8* knockdown on the development of neuronal dendrites, we performed morphological analysis of the layer II/III pyramidal neurons of somatosensory cortex 1 (S1). The layer II/III pyramidal neurons of S1 constitute the main excitatory neuronal subtypes in the neocortex and typically having an apical dendrite that branches out in an apical tuft that terminates in layer I [31]. Most scrambled shRNA-transfected neurons displayed a typical polarized dendritic arbor (Fig. 6a, b). In contrast, the *Chd8* shRNA significantly decreased the secondary branches and the total number and mean length of dendritic branches in neurons (Fig. 6c, d). The complexity of dendrites was also significantly decreased by *Chd8* shRNA constructs, revealed by Sholl analysis (Fig. 6e). These results indicated that CHD8 plays an essential role in neuronal morphogenesis in the mouse cerebral cortex.

### CHD8 deficiency impairs the migration of cortical neurons

**Fig. 7:**
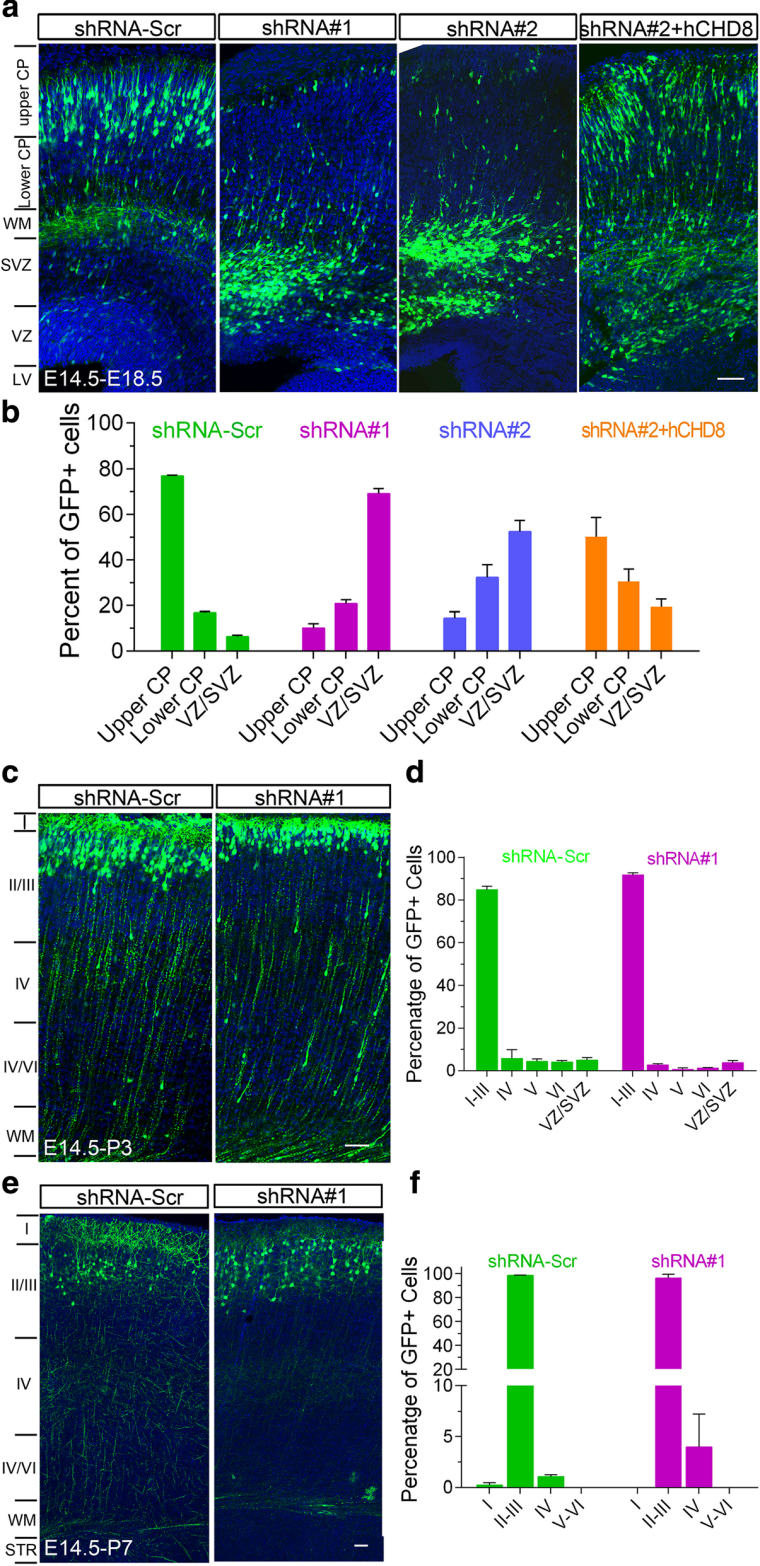
*Chd8* knockdown delays neuronal migration in vivo. **a** Representative images of coronal slices of E18.5 mouse brains that were transfected with GFP together with indicated *Chd8* constructs by in utero electroporation at E14.5. At E18.5, cortical neurons transfected with *Chd8*-shRNA constructs were misplaced in the VZ/SVZ instead of CP. Transfected cells were visualized by staining coronal slices with GFP antibody. Scale bar, 200 μm. **b** Quantitative analysis of the distribution of cortical neurons transfected with different constructs across three different zones in the somatosensory cortex: upper cp, lower cp, VZ/SVZ. Results are mean ± SEM from 6 to 12 sections (one per mouse) for each experiment. **c**, **d** By P3, cortical neurons migrated normally from the SVZ into the cortex in both control and Chd8 RNAi-transfected brains. Scale bar, 120 μm. **e**, **f** At P7, most transfected neurons were located in the layers II/III in both control and *Chd8* shRNA-transfected brains. Scale bar, 200 μm

Because of the expression of CHD8 in DCX-positive neural precursors, we examined whether its deficiency affected the radial migration of cortical neurons. The shRNA and EGFP constructs were co-electroporated into the developing mouse cortex at E14.5 using in utero electroporation (IUE) as previously described [27], and the distribution of EGFP-labeled cells in neocortex was examined at E18.5. While the majority of the EGFP-positive cells (76.8 ± 0.6%) migrated to the upper cortical plate (CP) when scrambled shRNA was co-expressed, only a small percentage of EGFP cells were observed in the upper CP when the *Chd8* shRNA constructs were co-expressed (shRNA#1, 10.1 ± 1.2%; shRNA#2, 14.4 ± 0.9%;) and the majority of labeled cells remained in the VZ/SVZ (Fig. 7a, b). Interestingly, the number of EGFP-positive cells that reached the upper CP was comparable between shRNA construct and scrambled controls at P3 and P7 (Fig. 7c–f). Accordingly, there are a comparable number of neurons that remained in the white matter (WM) and layers IV–VI. Together, these results indicate that deficiency of *Chd8* could cause a delay in radial migration of cortical neurons during early embryonic development, but this normalizes during postnatal development.

## Discussion

Genetic studies have provided strong evidence supporting an etiological role for genes encoding the proteins of epigenetic machinery in ASD [10, 34]. *CHD8* is one of the most prominent and repeatedly reported genes in this class [13]. In contrast to the well-characterized synaptic genes that are also strongly implicated in ASD, the molecular pathogenesis of ASD caused by deficiency of epigenetic machinery proteins such as *CHD8* remains to be thoroughly investigated. Through studies of *Chd8* deficiency in cultured rodent neurons or mutant mice by different experimental approaches, several groups have provided initial evidence supporting a causal link between a deficiency of CHD8 and neural cell proliferation, synaptic function, myelination, and ASD-like behaviors. The major findings from these studies and the current study are summarized in Table 1 [18, 20–25, 35, 36]. The results among these studies are not always consistent; in some cases, opposite findings are reported. For example, the decreased proliferation of neural progenitor cells is associated with *Chd8* deficiency using RNAi by in utero electroporation but the increased proliferation was reported in mice with germline mutation. Interestingly, a recent study of a new line of *Chd8* mutant mice reports sexually dimorphic phenotypes at molecular, synaptic function, and behavioral levels [24]. Another report also finds that *Chd8* deficiency, selectively in oligodendrocytes but not in neurons, results in myelination defects in mice. Similarly, different ASD-related behavioral phenotypes are also observed among different lines of *Chd8* mutant mice. Unfortunately, behavioral phenotypes have not been examined in oligodendrocyte-specific *Chd8* deficiency mice. Whether these discrepancies are due to different *Chd8* mutations in these lines of mutant mice or other experimental procedure-related issues are not immediately clear. Future studies of performing the experiments in parallel in different lines *Chd8* mutant mice are warranted.

**Table 1:** Summary of the findings from the current study and relevant literature

Reference(s)	Endogenous CHD8 expression data relating to the current study	Model system(s) and method(s) used for disrupting CHD8 expression	Findings caused by disrupting CHD8 expression relating to the current study
Current study	● Human (Brodmann region 19): CHD8 expressed in astrocytes (GFAP+) and in neurons (MAP2+, PV+, DCX+) by immunohistochemical stains ● Mice (whole brain lysates): CHD8 expression peaks at E16-E18, decreases substantially postnatally by qPCR and western blotting ● Mice (P3 brain sections and cultured neurons): CHD8 expression shows nuclear localization in MAP2+ neurons by immunostaining ● Mice (cultured neurons and glia): CHD8 is expressed in cultured neurons and to a lesser extent in cultured glia by western blotting	● Mice (in vitro)—cultured neurons transfected with *Chd8* shRNA constructs ● Mice (in vivo)—in utero electroporation at E14.5 with *Chd8* shRNA constructs	● Decreased axon length (both in vitro and in vivo) ● Reduced dendritic complexity (both in vitro and in vivo) ● Delayed neuronal migration (higher percentage of GFP+ cells in VZ/SVZ instead of CP) at E18.5 (in vivo), which normalized by P3
Durak et al. [18]	Mice (brain lysates): expression peaks at E12, then decreases during embryonic development to P2 by qPCR Human (DFC and MFC): expression peaks during early-mid fetal development and decreases from late fetal development to childhood by qPCR	Mice—in utero electroporation at E13 (for neuronal proliferation/migration studies) or E15 (for dendritic arborization studies) with *Chd8* shRNA constructs	● Increased neuronal migration (more GFP+ cells in CP instead of VZ/SVZ) at E16, accompanied by reduced proliferation, increased cell cycle exit, reduced mitotic activity, and premature Tuj1 expression ● Decreased dendritic arborization in upper cortical neurons from 5-month-old mice ● CHD8 mediates cortical neurogenesis via transcriptional regulation of cell cycle and Wnt signaling
Nishiyama et al. [20] and Katayama et al. [21]	Mice (whole embryos): CHD8 was expressed in ES cells (~E3.5), E8.5 embryos, E12.5 embryos, E16.5 embryos, and to a lesser extent in newborn pups by western blotting	Mice—two lines of germline haploinsufficient mice were generated (replacing 9 exons with loxP-neo cassette, or ∆exons 11–13, disrupting only the long protein isoform)	● Homozygous mutants show massive apoptosis at E7.5 and lethality ● At E14.5, haploinsufficient mice showed increased expression of early-fetal genes and decreased expression of mid-fetal genes
Platt et al. [22]	Mice (whole brains): expression profile from E10-P0, adult. Expression is highest at E10 and decreases over development by western blotting Mice (adult brain sections, somatosensory cortex): CHD8 shows nuclear localization and co-expresses with NeuN, PV, CNP1, and GFAP by immunostaining	Mice—germline haploinsufficient mice were generated by CRISPR/Cas9 introducing a 7 nucleotide deletion in exon 1, which disrupted the expression of both CHD8 isoforms	● Normal lamination and specification of neuronal cell types at P21 ● No difference in number of cortical progenitor cells or cell cycle length at E15.5
Zhao et al. [25]	Mice (P14 white matter tracts): CHD8 is expressed in oligodendrocytes and to a lesser extent in GFAP+ astrocytes by immunostaining Human (cerebellum): CHD8 expression in Sox10+ oligodendrocytes by immunostaining Mice (P14 cortical sections): CHD8 is expressed in NeuN+ neurons but not clearly in GFAP+ astroctyes or Iba1+ microglia by immunostaining	Conditional *Chd8* knockout mice (loxP sites surrounding exon 4) crossed with oligodendrocyte-specific Cre line	● Homozygous conditional knockout mice show lethality by P21 and myelination defects ● Homozygous conditional knockout mice show reduced proliferation of oligodendrocyte precursor cells in P1 spinal cord ● Dual requirement of CHD8 for chromatin landscape establishment and histone methyltransferase recruitment to promote CNS myelination and repair
Gompers et al. [35]	Expression analysis was not reported for different cell types or developmental time points	Mice—germline *Chd8* haploinsufficient mice were generated (deletion of exon 5)	● Increased proliferation of neuronal progenitors at E14.5 ● No gross lamination errors by P0 and P7
Wang et al. [36] CRISPR/Cas9-mediated heterozygous knockout of the autism gene *CHD8* and characterization of its transcriptional networks in neurodevelopment	N/A	CRISPR/Cas9-mediated heterozygous knockout of *CHD8* in iPSCs	● Genes involved in cell cycle, neuronal differentiation, and in neuronal projection development are dysregulated
Suetterlin et al. [23]	N/A	*Chd8*+/− mice (loxP sites on exon 3) crossed β-actinCre line	● *Chd8* heterozygous mice display increased total brain volume and showed volumetric increased of several brain regions, including cortical areas, hippocampus and parts of the cerebellum by high-solution MRI ● CHD8 controls the expression of ASD-associated axon guidance genes in the early postnatal neocortex
Jung et al. [24]	In both males and females, CHD8 protein was more abundant in the brain relative to other tissues, at embryonic stages relative to postnatal stages, and in non-crude synaptosomal fractions	*Chd8*+/N2373 K mice carrying a heterozygous Chd8 frame-shift mutation	● Distinct c-fos signals in male and female *Chd8*^+/N2373K^ brains under baseline and maternal-separation conditions ● Opposite changes in inhibitory synaptic transmission in the male and female *Chd8*^+/N2373K^ hippocampus

In this study, we have shown that CHD8 is expressed at high level in both NeuN-positive mature neurons and parvalbumin-positive interneurons and to a lesser extent in GFAP-positive astrocytes in human cerebral cortex. A previous study has found that the peak expression of CHD8 is between 13 and 19 weeks in the dorsolateral prefrontal cortex and 10–13 weeks of medial prefrontal cortex in humans [18]. In the developing mouse brain, we find that *Chd8/CHD8* expression peaks around embryonic days 16–18 and decreases to much lower levels by P21 by both mRNA and protein analyses. This finding is largely consistent with the reports of the development-specific expression of CHD8 in previous studies [18, 20, 22]. However, there are differences in the experimental designs among these studies. In our study, the *Chd8* expression study by real-time RT-PCR, RNA in situ hybridization, and western blot are performed in tissues from E16 day embryos to adult brains. In study by Nishiyama et al., the expression is examined by western blot and whole mount in situ in cells or tissues from embryonic stem (ES) cells as well as from E7.5 day embryos to adult mice [20]. The expression is high in ES and early embryonic stage, but a definitive peak is not seen. In other study by Platt et al. [22], real-time RT-PCR, western blot, and RNA in situ hybridization are used to examine the *Chd8* expression in brain tissues from E10 day embryos to adult mice. The highest expression is seen around E10–11 day embryos. In the study that the expression is examined in brain tissues from E12 to P2, the highest expression is at E12 [18]. With the caveats in the experimental designs, it is possible that the peak expression of *Chd8* is before E7 day because homozygous *Chd8* mutant are arrested before gastrulation because of massive apoptosis [20].

Consistent with previous report [18], we have also shown the evidence supporting the role of CHD8 in dendrite development of pyramidal neurons. The loss of CHD8 inhibits dendritic outgrowth and branch formation. In contrast to other reports, we have shown for the first time from both in vitro and in vivo studies that deficiency of CHD8 in developing mouse neurons inhibits axon growth and branch formation and results in a disruption of callosal axon projections to the contralateral cortex. The effect of CHD8 deficiency on the growth of axons is consistent with the report of dysregulation of genes implicated in axonal development in IPSC-derived neurons from human CHD8 patients [36, 37]. Together, our data demonstrate that CHD8 is important for normal dendritic and axonal development and that dysregulation of the process may contribute abnormal neuronal connectivity.

The peak expression in early and middle fetal development suggests a functional role of CHD8 in cortical development. Indeed, we found that *Chd8* knockdown results in a significant delayed migration of newborn cortical neurons examined at E18.5. This is opposite to the enhanced migration reported by Durak et al. using the similar approach but at different time points during the fetal development [18]. We performed in utero electroporation for *Chd8* knockdown at E14.5 and analyzed the cell migration at E18.5 while Durak et al. performed the in utero electroporation at E14 and analyzed at E16. Whether the different timing of CHD8 deficiency is responsible for the observed phenotypic differences remains to be determined. Because transcriptional regulation implicated in cortical development is dynamic and epigenetically modulated [38], it is also conceivable that CHD8 may have different roles at the different stages of fetal brain development. Interestingly, the delayed neuronal migration associated with knockdown of *Chd8* in our study is recovered at P3 and P7. This may suggest a catch-up period of the CHD8-deficient neurons after E18.5 days. Taken together, our findings support that CHD8 plays a critical role in migration of mitotic neurons as well as both dendritic and axonal growth in neocortex.

Human patients with *CHD8* mutations present with typical autistic behaviors and comorbidities including social deficits and communication difficulties, repetitive behaviors and interests, and cognitive delays [8, 13]. However, the findings from existing studies in model organisms have not provided a clear mechanistic link between genetic defects and clinical presentations [13, 21, 22, 25, 35, 39]. One of the notable features in some of the patients with *CHD8* mutations is relatively enlarged brain (macrocephaly) [13]. This feature is consistent with the findings of enlarged brains in a sub-set of idiopathic ASD cases during early postnatal brain development. Germline mutations of *Chd8* in zebrafish and mice have recapitulated the feature of enlarged brain size [13, 21, 22, 25, 35]. Accordingly, increased proliferation of neuronal progenitor cells and dysregulation of expression cell cycle genes are associated with germline line *Chd8* mutation [35]. Whether this is the underlying mechanism for macrocephaly associated with *CHD8* mutations and whether it is generalizable to the macrocephaly in other idiopathic ASD cases remain to be investigated. Notably, massive apoptosis is observed in E5.5 embryos carrying germline *Chd8* homozygous mutation [20] and somatic knockdown of *Chd8* by RNAi using in utero electroporation results in a decrease proliferation of neural progenitors [18]. These observations may indicate a different role of CHD8 at different developmental stages. More importantly, a critical question for future investigation is whether the cellular defects and enlarged brain size are responsible for ASD-like behaviors observed in *Chd8* heterozygous mutant animals.

In summary, CHD8 protein is highly enriched in neurons of developing neocortex in both human and mice and plays a critical role in regulating neuronal migration as well as growth of neurites. Our findings suggest that *CHD8* mutations in humans may also result in defects of neuronal migration and morphogenesis defects during a crucial stage of brain development and this impaired process may contribute to the pathophysiology of ASD.

## Additional file

Additional file 1: Validation of rescued constructs against shRNA-mediated knockdown of mouse *Chd8* in transfected neurons in vitro. (JPG 92 kb)

## Abbreviations

ASD: Autism spectrum disorder
CC: Corpus callosum
CHD8: Chromodomain helicase DNA-binding protein 8
CP: Cortical plate
hCHD8: Human CHD8 protein
IHC: Immunohistochemistry
IUE: In utero electroporation
qRT-PCR: Quantitative real-time RNA
SP: Sub-plate
SVZ: Subventricular zone
VZ: The ventricular zone
