# Supplementary figures and images for: Autism-associated CHD8 deficiency impairs axon development and migration of cortical neurons
Source: Mol Autism. 2018 Dec 19;9:65. doi: 10.1186/s13229-018-0244-2 (PMC6299922; doi:10.1186/s13229-018-0244-2)

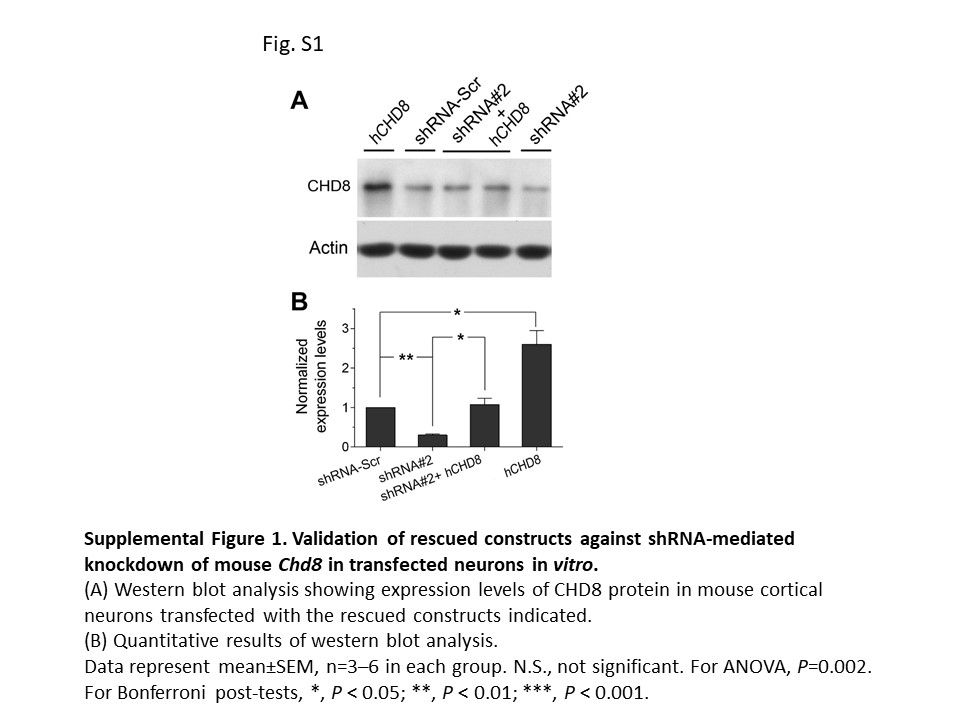

Supplement: Supplementary file 1 — Validation of rescued constructs against shRNA-mediated knockdown of mouse Chd8 in transfected neurons in vitro. (JPG 92 kb) [file 13229_2018_244_MOESM1_ESM.jpg]
